# Selected parameters of epidermal barrier in juveniles with type 1 diabetes correspond with the severity of diabetes – an observational study

**DOI:** 10.3389/fendo.2025.1709604

**Published:** 2025-11-25

**Authors:** Klaudia Bogusz-Górna, Adriana Polańska, Aleksandra Dańczak-Pazdrowska, Ryszard Żaba, Piotr Fichna, Andrzej Kędzia

**Affiliations:** 1 Department of Pediatric Diabetes, Auxology, and Obesity, Poznan University of Medical Sciences, Poznan, Poland; 2 Department of Dermatology, Poznan University of Medical Sciences, Poznan, Poland

**Keywords:** children, adolescents, type 1 diabetes, epidermal barrier, transepidermal water loss, TEWL, epidermis hydration, corneometry

## Abstract

**Background:**

Our study aimed to evaluate the epidermal barrier function in children with type 1 diabetes. To the author’s knowledge, no studies have been conducted on epidermal barrier parameters, including TEWL and measurement of epidermal hydration, in children and adolescents with type 1 diabetes.

**Methods:**

One hundred sixty children and adolescents aged 6-18, 125 patients with type 1 diabetes, and 35 healthy volunteers participated in the study: a detailed clinical evaluation, transepidermal water loss (TEWL) measurement, and epidermal hydration (corneometry) were carried out.

**Results:**

Poor metabolic control in type 1 diabetes, higher HbA1c(%), and more frequent hyperglycemia impact TEWL and epidermis hydration. Also, the level of BF(%) correlated positively with TEWL.

**Conclusions:**

Extended supplementary tests – the assessment of TEWL and corneometry – could be included in the periodic examinations of children and adolescents with type 1 diabetes mellitus. Clinicians should always pay attention to dry skin in children with diabetes, and noninvasive examination (TEWL measurement and corneometry) may allow us to isolate a group at risk of neuropathy or the development of the diabetic foot. Further tests enabling detailed assessment of the usefulness of TEWL measurements and corneometry are needed.

## 1 Introduction

The epidermal barrier is primarily formed by the stratum corneum (SC), which consists of corneocytes surrounded by lamellar lipid structures (1, 2). Although not yet fully understood, several mechanisms contribute to epidermal barrier dysfunction in diabetes. Impairment of microvascular blood flow and sweat gland receptor activity may represent some of the earliest manifestations of neuropathy, resulting in dry skin, diminished or absent perspiration, and the development of cracks and fissures that facilitate skin infections and diabetic foot complications (3, 4). Assessment of the epidermal barrier and the biophysical properties of the SC commonly involves corneometry and transepidermal water loss (TEWL) measurements. Both TEWL and epidermal hydration are influenced by skin and epidermal thickness, and therefore vary depending on anatomical location. TEWL reflects the permeability of the epidermal barrier by measuring the rate of water evaporation from the skin Surface (4, 5). Lower TEWL values indicate an intact and well-functioning barrier with low permeability, whereas higher values signify greater barrier impairment (6) Corneometry is a well-established technique for evaluating epidermal water content due to its high reproducibility, short measurement time, ease of use, and relatively low cost (7). The corneometer operates by measuring electrical capacitance, which increases in proportion to tissue hydration within a depth of approximately 10–20 μm (8). The results are expressed in arbitrary units, typically ranging from 0 to 130, with higher values indicating better epidermal hydration (7). The aim of this study was to evaluate the function of the epidermal barrier in children with type 1 diabetes in relation to clinical and laboratory parameters.

## 2 Materials and methods

**Figure 1:**
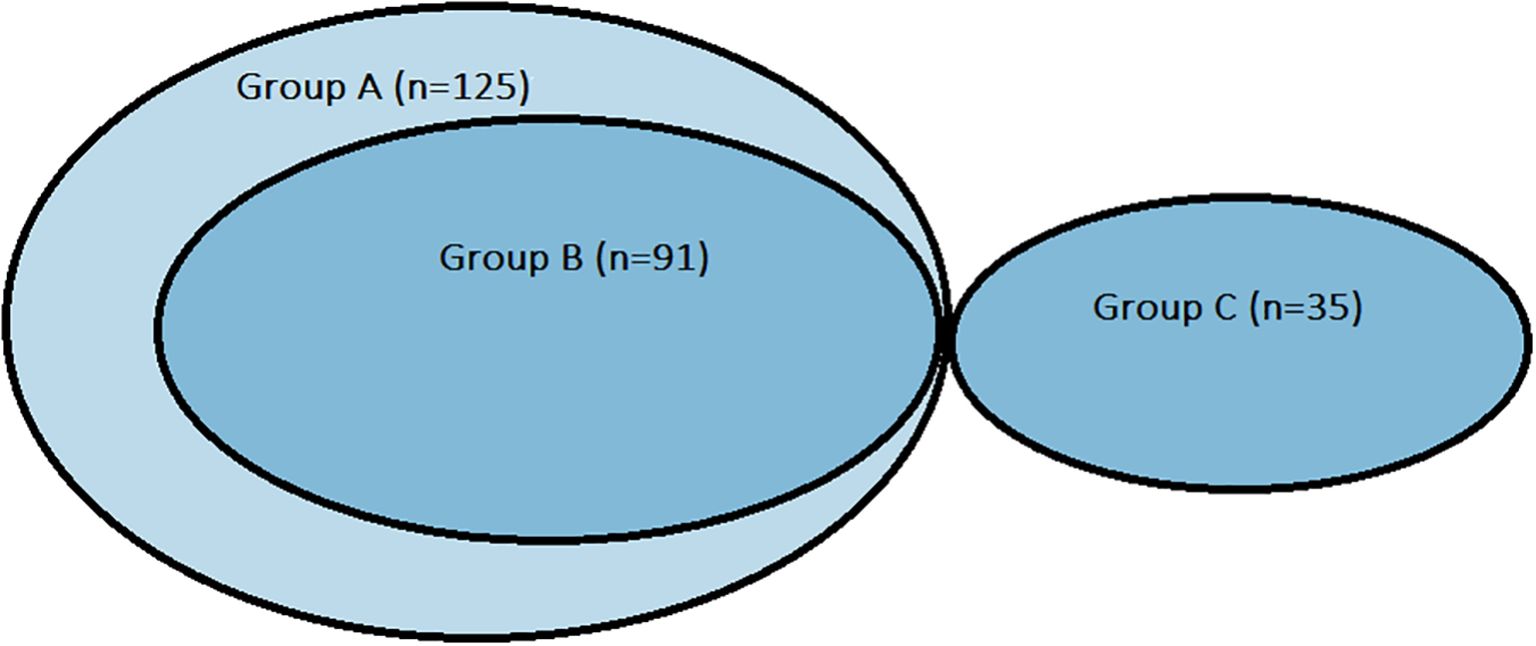
Chart presenting groups distribution. Group A (n=125): all patients with diabetes participating in the study. Group B (n=91): patients with type 1 diabetes selected from Group A comparable with control group regarding sex and age. Group C (n=35): control group.

The results of the presented study are part of a larger research project, along with those presented in Non-invasive detection of early microvascular changes in juveniles with type 1 diabetes (9, 10). We recruited individuals ranging from middle childhood to adolescence with type 1 diabetes from the Department of Pediatric Diabetes, Auxology, and Obesity and the outpatient ambulatory Childhood Diabetes Clinic at Poznan University of Medical Sciences (Poland). The study was designed and realized according to the principles of the Declaration of Helsinki as revised in 2008 and approved by the Local Bioethics Committee of Poznan University of Medical Sciences (185/19). The study group included patients with type 1 diabetes diagnosed according to WHO criteria [fasting plasma glucose values of ≥ 7.0 mmol/L (126 mg/dl), 2-h post-load plasma glucose ≥ 11.1 mmol/L (200 mg/dl), HbA1c ≥ 6.5% (48 mmol/mol); or a random blood glucose ≥ 11.1 mmol/L (200 mg/dl) in the presence of signs and symptoms] (11) during insulin therapy for 7.09 ± 3.41 years (min 1 year, max 14.3 years). The enrolled patients ranged from 6 to 18 years of age. The control group included healthy children aged 6-18: siblings of children in the study group. Participants and their parents gave informed consent. The study aimed to investigate the isolated effect of diabetes within the study cohort while minimizing the potential impact of known confounding factors. Consequently, the control group was composed of the patients’ siblings, as they share similar genetic and environmental backgrounds with their counterparts diagnosed with diabetes. Children with active infections or cutaneous lesions in the areas designated for testing were excluded. Participants with current atopic dermatitis were also excluded, and all subjects were instructed to refrain from applying emollients or barrier creams to the examined skin for at least 24 hours prior to assessment. A total of 160 participants completed the study, including 125 individuals with type 1 diabetes and 35 healthy siblings who served as controls (**Figure 1**). The study protocol comprised a detailed medical history with supplementary analysis of medical records, physical examination, and assessment of transepidermal water loss (TEWL) and corneometry. Anthropometric measurements included height [stadiometr Holstein (United Kingdom)], weight [electronic scales WPT 150, Radwag (Poland)] with the calculation of BMI-SDS (12, 13), bioelectrical impedance analysis [Tanita MC-980 MA (Tanita Corporation, Tokyo, Japan)] defined as body fat mass percentage – BF% and skinfolds thickness [Holtain Skinfold Caliper (United Kingdom)] of the abdominal area, arm above triceps and subscapular region. Capillaroscopy and photoplethysmography were performed at the Department of Dermatology, Poznań University of Medical Sciences, and the results are presented in a previously published paper (9). In accordance with the 2018 recommendations of the European Group on Efficacy Measurement and Evaluation of Cosmetics and Other Products (EEMCO), corneometry and TEWL measurements were conducted to obtain a comprehensive assessment of skin hydration (5). In accordance with literature data, the flexural surface of the upper limb (antecubital fossa) was selected as the measurement site. TEWL and epidermal hydration were assessed within representative areas following established guidelines, on macroscopically intact skin (7). To perform the TEWL measurement, we used the Tewameter TM 300 from Courage-Khazaka (Köln, Germany), connected to the Cutometr MPA 580 adapter. The TEWL measurement was performed under controlled conditions, including stable ambient temperature and relative humidity. Prior to assessment, participants underwent a 20-minute acclimatization period to ensure skin equilibrium with the environment. Measurements were taken using a closed-chamber probe in accordance with the manufacturer’s instructions, and each result represented the mean of three consecutive readings obtained from the same site. Epidermal hydration was evaluated using the Corneometr CM 825 device from Courage-Khazaka (Köln, Germany) connected to the Cutometr MPA 580 adapter, and the final value was calculated as the mean of three one-second measurements performed under identical conditions. The study was carried out according to the recommendations of EEMCO and the European Society of Contact Dermatitis (14–16). All measurements were conducted at the same time of day, between 11:00 and 13:00, to minimize the potential influence of circadian variation on skin parameters.

### 2.1 Statistical analyses

Statistical analyses were performed using IBM SPSS Statistics version 23. The Kolmogorov–Smirnov test was applied to assess the normality of quantitative variables. Descriptive statistics were calculated accordingly: for normally distributed data, the mean and standard deviation, as well as minimum and maximum values, were reported; for non-normally distributed data, the median along with minimum and maximum values was presented. For variables that did not conform to a normal distribution, non-parametric tests were applied, whereas parametric tests were used for normally distributed variables. Depending on the scale of measurement and distribution characteristics, the following tests were employed to compare study groups and assess relationships between variables: Student’s t-test, Mann–Whitney U test, one-way analysis of variance (ANOVA), Kruskal–Wallis test, χ² test, and Fisher’s exact test. Specific references to the tests applied for each analysis are provided in the *Results* section. Correlation analyses were conducted using the Pearson correlation coefficient (r) for quantitative variables with a normal distribution. For variables that were non-normally distributed, as well as for dichotomous or ordinal variables, Spearman’s rank correlation coefficient (ρ) was applied. Ordinal variables were coded so that higher ranks corresponded to greater levels of the feature, while for dichotomous variables, higher ranks indicated the presence of the feature. A p-value of 0.05 was considered statistically significant.

**Figure 2:**
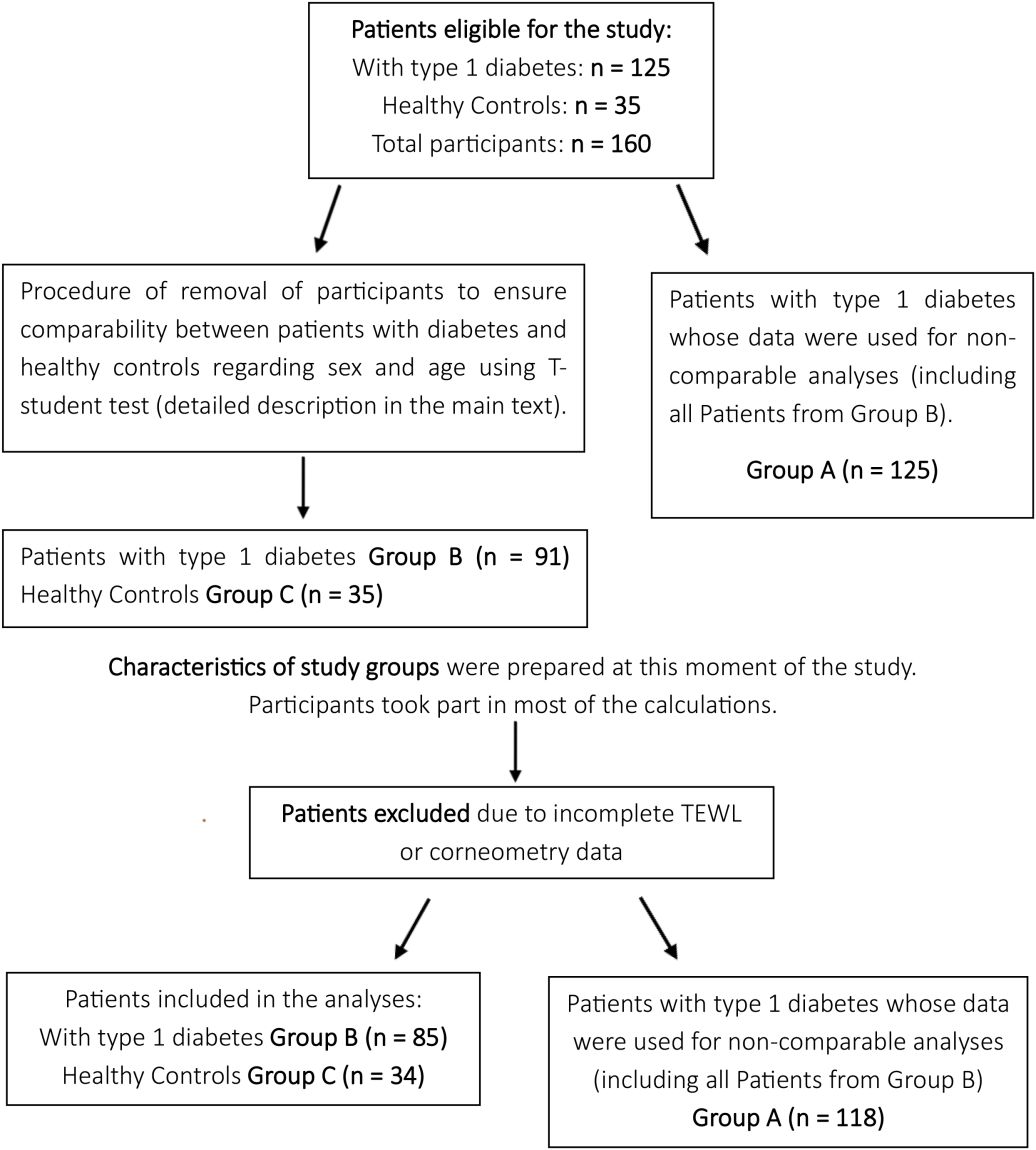
Flowchart illustrating participants distribution.

As a result of selecting siblings for the control group, the study groups differed in size, sex, and age, with control participants being younger and predominantly male. To minimize the potential confounding effects of age and sex, a subgroup of patients with diabetes (Group B) was created to be comparable with the control group (Group C) with respect to these variables. Given the small size of the control group, the adjustment was made by modifying the composition of the group of patients with diabetes (**Figure 2**).

The procedure for removing subjects from the database was conducted by an independent statistician, who had access only to the encoded database and was initially blinded to the study’s objectives. Individuals were excluded from the study group to eliminate differences in sex and age between the study and control groups, while minimizing data loss. This was achieved by gradually removing data from female participants and the oldest patients in the study group. Group comparisons were performed using the t-test.

As a result, a subgroup of patients with diabetes (Group B) was established, matched to the control group (Group C) with respect to sex and age, for comparative analyses. Although this approach carries potential bias, the selection process was fully independent of the researchers’ influence. Consequently, three groups were defined: Group A (n = 125), comprising all patients with diabetes, whose data were used for analyses not requiring a control comparison; Group B (n = 91), a subset of Group A matched to the control group for sex and age; and Group C (n = 35), the control group of healthy children without diabetes.

## 3 Results

### 3.1 Characteristics of study groups

The study group included patients with type 1 diabetes who had been receiving insulin therapy for 7.09 ± 3.41 years (range: 1–14.3 years). All characteristics are summarized in **Table 1**. Two statistically significant differences were observed between Groups B and C: patients with diabetes exhibited significantly greater arm skinfold thickness. At the other two skinfold measurement sites, no statistically significant differences were detected, although there was a trend toward higher values in Group B. Comorbidity analysis revealed that autoimmune thyroiditis was significantly more frequent in patients with diabetes. Groups A and C were not compared; data from Group A were utilized solely for analyses that did not require a control group.

**Table 1:** Characteristics of studied groups.

Characteristics	Group A (n=125) mean ± SD min/max	Group B (n=91) mean ± SD min/max	Group C (n=35) mean ± SD min/max	Comparison: group B with C (test used)
Age (years)	13.32 ± 2.90 min 6.7 - max 18	12.3 ± 2.64 min. 6.7 - max. 16.3	11.15 ± 3.43 min. 6 - max. 18	*p* = 0.079 (▪)
Gender: female (%)	71 (56.80%)	51 (56%)	16 (45%)	*p* = 0.325 (*)
Diabetes duration (years)	7.09 ± 3.41 min 1 - max 14.3	6.66 ± 2.93 min. 2.09-max. 14.2	–	–
BMI-SDS	0.14 ± 0.81 min (-1.6) - max 2.3	0.04 ± 0.73 min (-1.6) - max 1.7	0.14 ± 1.04 min(-1.39) - max 2.5	*p* = 0.590 (▪)
BF(%)	22.19 ± 5.63 min 8.4 - max 39.6	21.82 ± 5.17 min 8.4 - max. 33.4	21.36 ± 7.03 min 10.1 – max 34.6	*p* = 0.785 (▪)
Skinfold thickness: arm (mm)	14.44 ± 8.3 min 2 - max 36	15.08 ± 6.46 min 2 - max. 30	11.30 ± 5.42 min 4 - max 22	*p* = 0.017 (▪)
Skinfold thickness: abdomen (mm)	15.46 ± 6.65 min 2 - max 38	13.4 ± 7.91 min 2 - max 36	10.20 ± 7.36 min 1 - max 29	*p* = 0.102 (▪)
Skinfold thickness: back (mm)	12.87 ± 6.84 min 1 - max 38	11.91 ± 5.78 min 4 - max 28	9.75 ± 6.90 min 1 - max 30	*p* = 0.151 (▪)
HbA_1c_ 3 months (%)	7.47 ± 1.77 min 5 - max 22	7.12 ± 1.91 min 5.8 - max 22	–	–
DID (IU/kg)	0.8 ± 0.17 min 0.39 - max 1.23	0.79 ± 0.17 min 0.41 - max 1.19	–	–
CSII	95 (76%)	70 (77%)	–	–
Retinopathy	0 (0%)	0 (0%)	–	–
Albuminuria	3 (2.40%)	1 (1.10%)	–	–
Neuropathy	2 (1.60%)	1 (1.10%)	–	–
Hypertension	10 (8%)	5 (5.50%)	–	–
Dyslipidemia	52 (41.60%)	40 (43.95%)	–	–
Autoimmune thyroiditis	32 (25.60%)	26 (28.60%)	0/0%	p <0.001 (*)
Celiac disease	14 (11.20%)	5 (5.50%)	1/2.90%	*p* = 0.177 (^)
Asthma	11 (8.80%)	11 (12.10%)	1/2.90%	*p* = 0.281 (^)
Epilepsy	0 (0%)	9 (9.90%)	1/2.90%	*p* = 0.321 (^)
Vitiligo	5 (4%)	0 (0%)	1/2.90%	*p* = 0.477 (^)

SD, standard deviation; min, minimum; max, maximum; BMI-SDS, body mass index standard deviation score; BF (%), body fat mass percentage; HbA1c, glycated hemoglobin – the result obtained during last three months; DID (IU/kg), daily insulin dose in units per kilogram; CSII, continuous subcutaneous insulin infusion; *p* – level of significance. (▪) student t-test (*) chi-square (χ^2^)test (^) Fishers exact test.

Group A (n=125) represents all of the patients with diabetes; we used their data for non-control statistical analyses; Group B (n=91) includes patients with type 1 diabetes selected from Group A, comparable with control group regarding sex and age. Group C (n=35) is the control group.

### 3.2 TEWL results

The mean TEWL values were measured in 118 patients from Group A, 85 patients from Group B, and 34 children from the control group (Group C). Participants with missing TEWL data were excluded from these analyses. TEWL values ≥25 g/m²/h, indicative of epidermal barrier impairment, were observed in two children with diabetes. Detailed TEWL results are provided in the **Supplementary Material** (**Supplementary Tables S1**, **S2**).

No statistically significant difference was observed in mean TEWL between Group B (children with diabetes; 10.96 ± 5.27 g/m²/h) and Group C (controls; 10.43 ± 2.56 g/m²/h; p = 0.884, Mann–Whitney U test). The mean TEWL value in Group A was 10.95 ± 5.02 g/m²/h.

**Figure 3:**
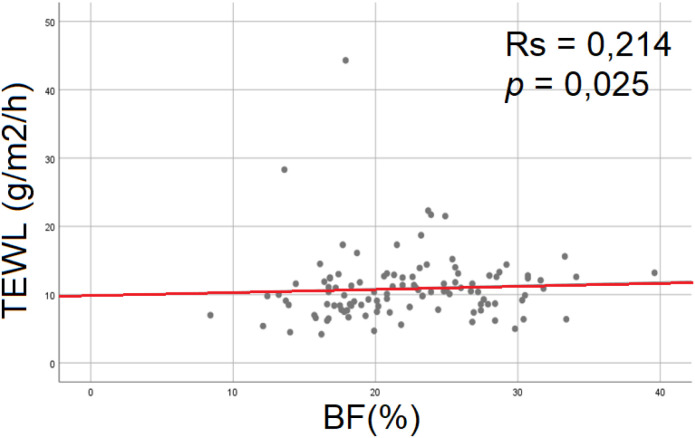
Correlation between TEWL and BF(%) in group A - the level of BF(%) correlated positively with TEWL. RS – Spearman’s correlation coefficient, TEWL, transepidermal water loss; BF(%), body fat mass.

Correlation analyses between TEWL and clinical parameters in Group A revealed a positive association with body fat percentage (BF%), indicating that children with higher adipose tissue content had higher mean TEWL values (**Figure 3**). No significant correlations were found between TEWL and age, BMI-SDS, or skinfold thickness measurements (**Supplementary Tables S1**, **S2**).

**Figure 4:**
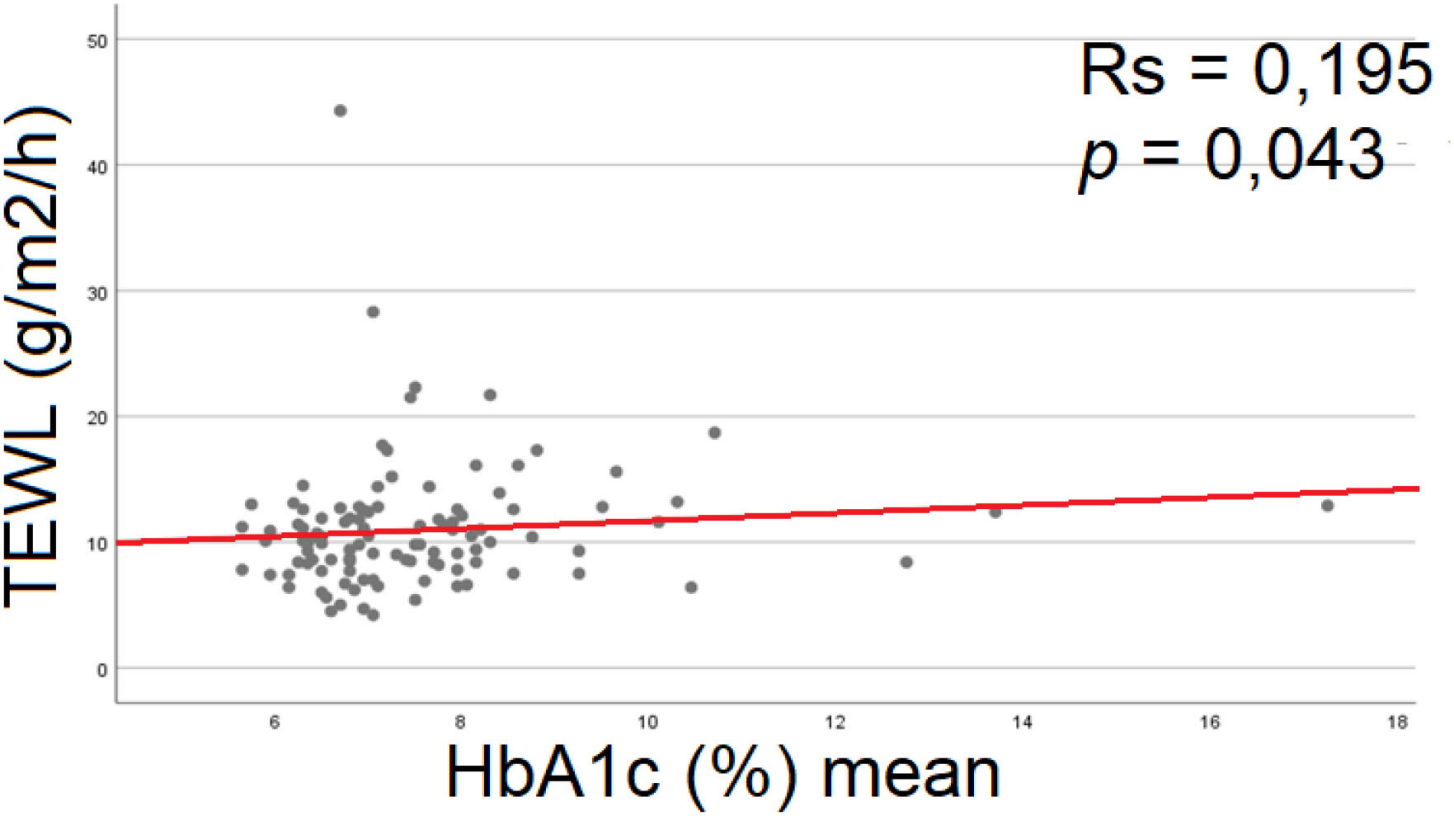
Correlation between TEWL and HbA1c (%) mean in group A - higher mean TEWL values characterized patients with higher mean HbA1c. RS, Spearman’s correlation coefficient; TEWL, transepidermal water loss; BF(%), body fat mass; HbA1c(%) –percentage of glycated hemoglobin.

Among the metabolic control parameters, a single statistically significant association was observed: patients with higher mean HbA1c levels exhibited higher mean TEWL values (**Figure 4**). This indicates that poorer metabolic control, as reflected by elevated glycated hemoglobin, is associated with increased transepidermal water loss and, consequently, impaired epidermal barrier function.

No statistically significant associations were observed between mean TEWL values and clinical parameters related to insulin therapy, disease duration, frequency of hypo- or hyperglycemic episodes, or the presence of microvascular complications. Interestingly, patients with asthma exhibited significantly lower mean TEWL values, although the p-value was marginal (p = 0.050). Detailed results are presented in the **Supplementary Material** (**Supplementary Table S3**). This finding is further explored in the *Discussion* section.

### 3.3 Corneometry results

Corneometry measurements were performed in 118 patients from Group A, 85 patients from Group B, and 34 children from the control group (Group C). Participants with missing corneometry data were excluded from the analyses. The results are summarized in **Table 2**. No statistically significant difference in corneometry values was observed between Groups B and C (Student’s t-test, p = 0.507).

**Table 2:** Corneometry results.

GROUP (n)	Corneometry (U)		
	Mean ± SD	Min.	Max.
Group A (n = 118)	34,45 ± 10,66	10,93	79,10
Group B (n = 85)	34,07 ± 9,72	6,0	61,5
Group C (n = 34)	33,07 ± 6,21	22,2	48,6

SD, standard deviation.

**Figure 5:**
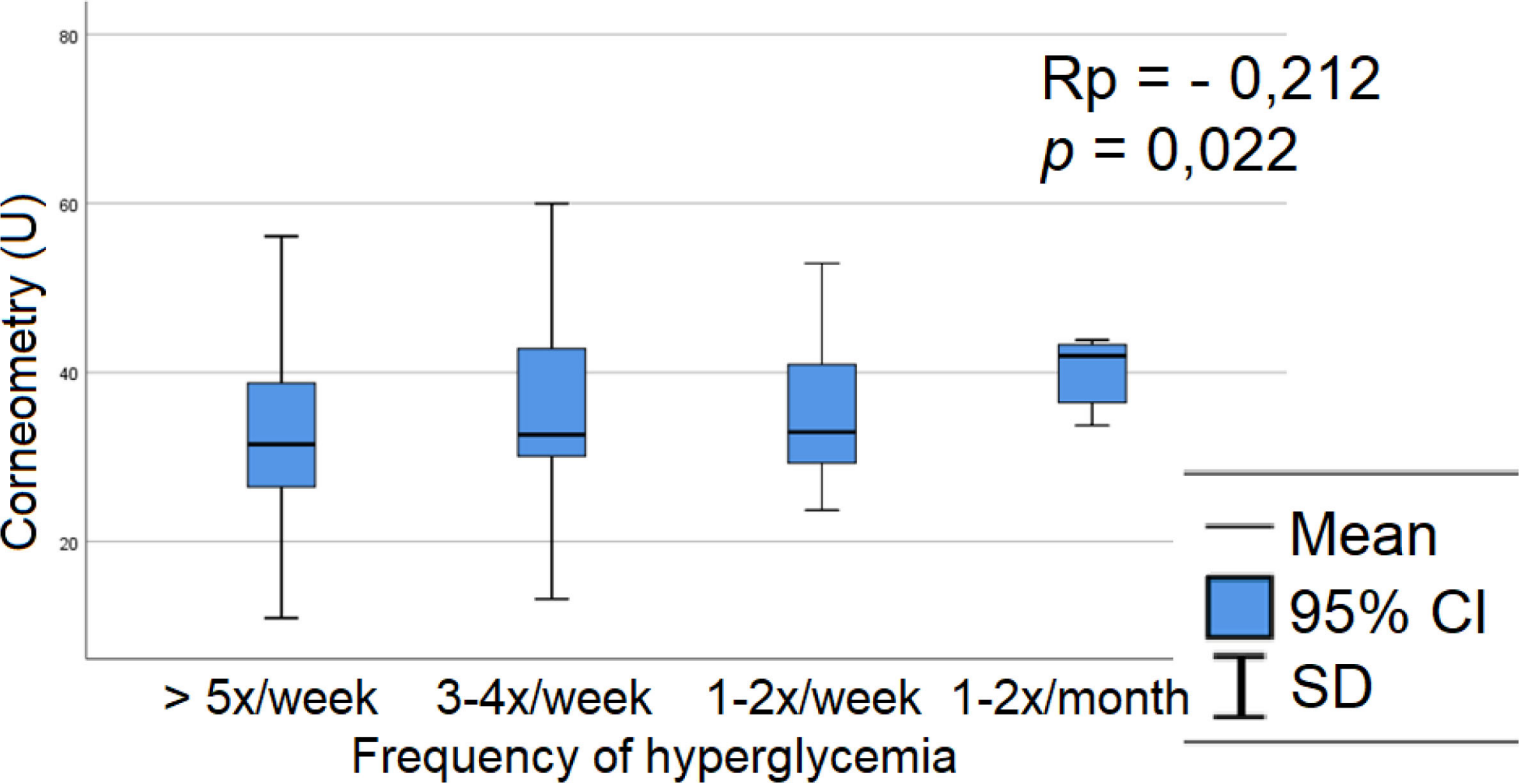
Relation between epidermis hydration and frequency of hyperglycemia in group A - The frequency of hyperglycemia correlated negatively with corneometry values, patients who experience hyperglycemia above 250mg/dl more often have a lower level of epidermal hydration. Rp, Pearson correlation coefficient; SD, standard deviation.

Within Group A, the relationship between corneometry measurements and clinical parameters, including insulin therapy, disease duration, frequency of hypo- or hyperglycemic episodes, and microvascular complications, was analyzed. Among these parameters, a single statistically significant association was identified: the frequency of hyperglycemia correlated negatively with corneometry values, indicating that patients who more frequently experienced hyperglycemia above 250 mg/dL had reduced epidermal hydration (**Figure 3**). As continuous glucose monitoring was not routinely used in this cohort, a frequency scale for hypo- and hyperglycemic episodes was developed based on glucometer readings. Patients were categorized into four groups according to the frequency of hypo- and hyperglycemia (**Figure 5**). Detailed corneometry results in relation to clinical parameters are provided in the **Supplementary Material** (**Supplementary Table S4**).

**Figure 6:**
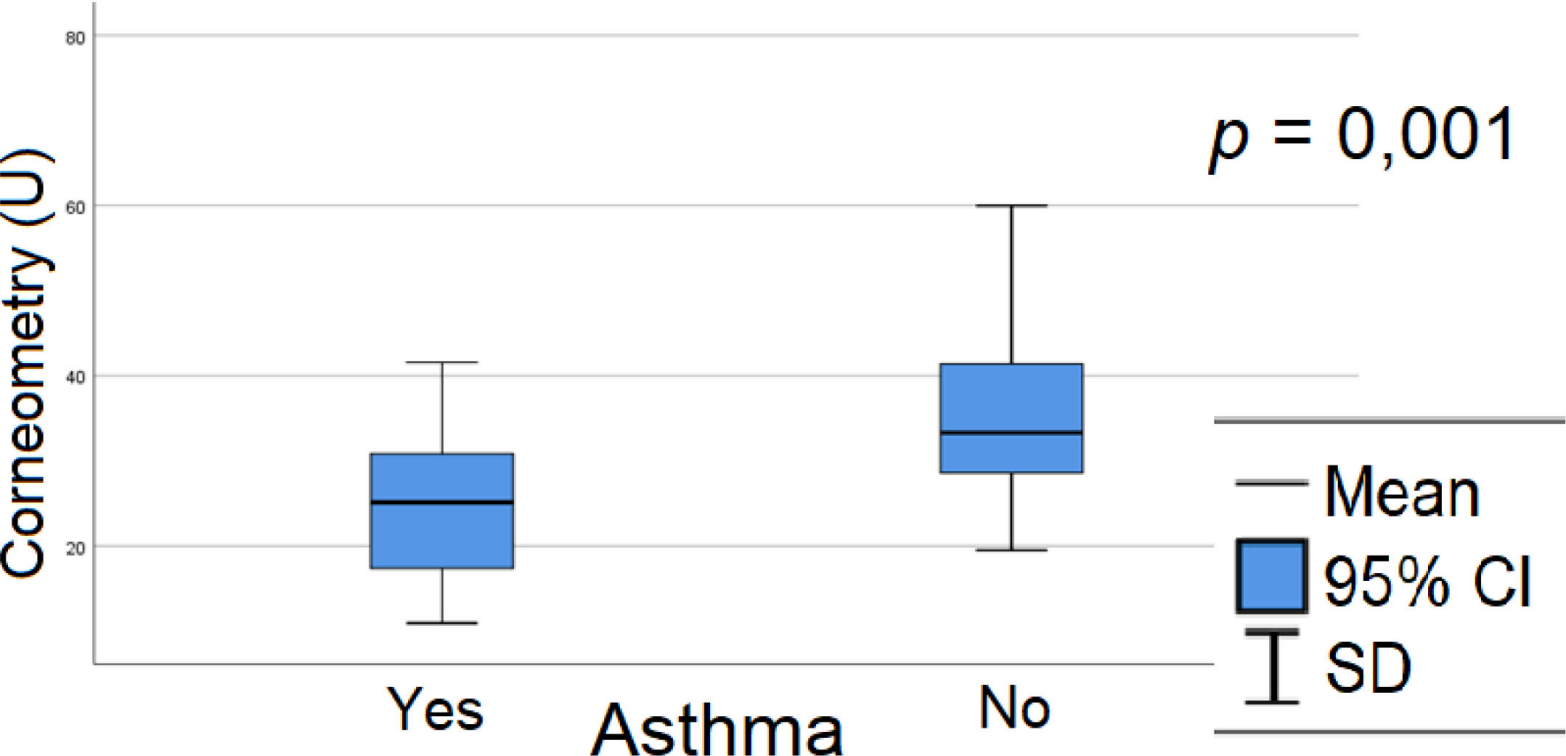
Relation between epidermis hydration and asthma in group A - patients with asthma had lower corneometry results, which means worse epidermal barrier hydration. Rp, Pearson correlation coefficient; SD, standard deviation.

Among comorbidities, a single statistically significant association was observed: patients with asthma exhibited lower corneometry values (**Figure 6**), indicating reduced epidermal hydration and suggesting a potential influence of atopy. No significant relationships were found between corneometry measurements and autoimmune thyroiditis, celiac disease, or vitiligo (**Supplementary Table S5**).

## 4 Discussion

Corneometry and TEWL measurements are well-established research methods in dermatology and cosmetology, and they are also widely used to assess the effects of damaging factors on the skin as well as the efficacy of protective interventions (15, 17). The correct TEWL value varies depending on the measurement site due to the difference in the thickness of the stratum corneum and skin vascularity. Usually, the value range is 1–15 g/m2/h, and TEWL above 25 g/m2/h is considered abnormal, typical for damaged skin (8). The corneometry result below 30 units characterizes dry skin; the measurement of epidermal hydration between 30 and 40 units indicates moderate skin dryness. Values ​​providing proper skin hydration are above 40 units (18). These two parameters are typically correlated, with higher TEWL values corresponding to lower corneometry readings. However, the reference ranges mentioned above apply to healthy adults, as no established ranges are available for children. It is important to note that the structure and function of the epidermis differ between adults and young children, reflecting the gradual maturation of the epidermal barrier with age. Most studies comparing epidermal barrier structure and function focus on adults and infants during the first year of life, demonstrating significant differences between these age groups (19, 20). Mack et al. demonstrated that the maturation of the epidermal barrier continues until at least the age of four, and that children’s skin does not achieve full maturity during this period (21). Kong et al. evaluated the biophysical properties of the skin in Chinese children aged 1, 2, 3, 4, 5, and 10 years, as well as in their mothers. They reported that TEWL values reach adult levels by approximately five years of age and remain stable thereafter. In contrast, corneometry measurements were significantly lower in children across all age groups compared to adults, including ten-year-old children (22). Walters et al. reported similar findings (23).

We examined children over six years of age. The average corneometry values in all groups did not exceed 40 units. Only 11.8% of children in the control group and 25% of patients with diabetes had corneometry values above 40 units, with no statistically significant difference between the groups. TEWL values exceeding 25 g/m²/h were observed in only two patients with diabetes. In both study groups, mean TEWL values remained below 11 g/m²/h, and no significant differences were detected between groups. These findings are consistent with the observations reported by Kong et al. (22) These results suggest that adult reference standards may be applicable for TEWL analysis in the children studied. However, when assessing epidermal hydration using a corneometer in children under ten years of age, caution should be exercised in interpreting values below 40 units as abnormal. Applying adult norms would imply that most children in both study groups had objectively dry skin, which may not accurately reflect their physiological state.

To the authors’ knowledge, no studies have specifically investigated epidermal barrier parameters, including TEWL and epidermal hydration, in children and adolescents with type 1 diabetes. Existing publications predominantly focus on adults, often with type 2 diabetes, long disease duration, and multiple complications (24–27). These differences limit the comparability of epidermal barrier parameters between children with relatively short disease duration and the adult populations studied. Nonetheless, the findings reported by previous researchers support the utility of TEWL and corneometry for assessing epidermal barrier hydration and its potential relationship with diabetic complications.

The impact of diabetes on the structure and function of the epidermal barrier has been extensively characterized in animal models. Okano et al. reported that diabetic mice exhibited increased epidermal barrier permeability, altered distribution of zonula occludens proteins, structural changes, and a reduction in the number of basal epidermal cells. Additionally, there was an increase in the number of corneocytes accompanied by reduced sensitivity to mechanical stress, as well as disrupted expression of keratins and loricrin. These alterations were ameliorated by insulin treatment, suggesting that both insulin deficiency and hyperglycemia significantly influence keratinocyte differentiation and proliferation, potentially representing a primary mechanism underlying epidermal barrier impairment in patients with diabetes (28). Sakai et al. examined streptozotocin-induced diabetic mice used as a type 1 diabetes model. They confirmed the results obtained in studies on adult patients with diabetes (mainly type 2), proving the influence of diabetes and acute hyperglycemia on the regulation of epidermal SC hydration (29, 30).

Park et al. investigated TEWL and corneometric measurements of epidermal hydration, identifying a relationship between reduced stratum corneum hydration and impaired epidermal regenerative capacity, indicative of homeostatic disturbances that correlated with HbA1c levels. In rats with chronic hyperglycemia, lipid synthesis in the stratum corneum was significantly reduced, while circulating advanced glycation end products (AGEs) and skin expression of AGE receptors were markedly increased. Additionally, antimicrobial defense mechanisms of the skin were impaired. Despite these changes, no increase in TEWL was observed as a consequence of chronic hyperglycemia (31).

Yosipovitch et al. evaluated epidermal hydration using a corneometer in 238 patients with type 1 diabetes under 30 years of age and in 80 healthy controls. No statistically significant difference in epidermal hydration was observed between diabetic patients and healthy volunteers (32), consistent with our findings. In contrast, Seung Hon Han et al. reported differing results in a study involving 42 adult patients with diabetes (4). The study assessed TEWL as an indicator of epidermal barrier function in patients with diabetes and its association with diabetic sensorimotor polyneuropathy (DSPN) and peripheral autonomic neuropathy (PAN), compared with healthy controls. TEWL was measured at multiple anatomical sites on the distal portions of the upper and lower limbs. Regardless of DSPN or PAN status, TEWL values on the hands and feet were significantly lower in the diabetic group than in controls, whereas values on the forearm and lower leg did not differ between groups. Within the diabetic cohort, participants with autonomic polyneuropathy exhibited reduced TEWL on the fingers, soles, and toes compared to patients without neuropathy. These findings suggest that epidermal barrier parameters deteriorate with age and diabetes duration, and that abnormal TEWL and corneometry values may represent early indicators of developing neuropathy.

In the cited studies, patients with type 1 diabetes were considerably older than those in our analysis, and most participants had type 2 diabetes with longer disease duration, multiple microvascular complications, and measurements taken at anatomical sites not directly comparable to those in our study. In the present study, TEWL and stratum corneum hydration were assessed on macroscopically intact skin at the flexural surface of the upper limb (antecubital fossa) in representative areas, following established guidelines as detailed in the *Methods* section. No statistically significant differences were observed between children with diabetes and healthy control subjects, demonstrating strong concordance with findings reported by other researchers (4, 27, 32). In the early stages of the disease, compensatory mechanisms may still preserve epidermal homeostasis, resulting in values comparable to those in healthy controls. A possible explanation is the relatively short disease duration in our study population, which may not have been sufficient to induce distinct alterations in skin barrier function. Additionally, variability in individual skincare routines and environmental exposure could have contributed to minimizing group differences.

We observed a positive correlation between increased adipose tissue content and TEWL (p = 0.025), as illustrated in **Figure 3**, whereas no such relationship was found for BMI-SDS. Similarly, Nino et al. reported higher TEWL values in children with obesity, although no direct correlation with BMI was identified (33). Increased subcutaneous fat thickness compared to non-obese individuals may contribute to elevated resting body temperature (4, 34). Although testing conditions were standardized for all participants, the contribution of sweat production to elevated TEWL in individuals with higher adipose tissue cannot be excluded. Excessive skin fat tissue expansion may also impair the barrier function of the skin epidermis, with mechanical stretching of the skin due to obesity potentially contributing to skin inflammation by impairing the epidermal barrier function and pre-deposition of keratinocytes under an activation state (35–37). Furthermore, the obesity-related reduction of keratins and desmosome structural components may cause skin fragility and contribute to skin barrier dysfunction (37).

Additionally, we observed a correlation between TEWL and long-term glycemic control: patients with higher mean HbA1c over the preceding year exhibited higher TEWL values (**Figure 4**). No such association was found for the three-month mean HbA1c (**Supplementary Table S2**), suggesting that chronic hyperglycemia has a greater impact on epidermal barrier permeability. In corneometric measurements, lower epidermal hydration values were observed in patients who experienced hyperglycemia more frequently (**Figure 5**). These findings (**Figures 3**–**5**) suggest a relationship between hyperglycemia, chronic glycemic control, and the biophysical properties of the epidermal barrier in children with diabetes. Insulin and IGF-1 receptors are present on keratinocytes. The proper function of insulin and insulin-like growth factor 1 (IGF-1) is crucial in maintaining epidermal homeostasis. Hyperglycemia reduces autophosphorylation of the ligand-induced IGF-1 receptor. Consequently, the physiological effects of insulin and IGF-1 on glucose uptake, proliferation, and differentiation of keratinocytes are impaired. Hyperglycemia and impaired insulin signaling lessen glucose utilization by skin keratinocytes and disturb their normal proliferation, migration, maturation, and differentiation (11). In addition, hyperglycemia decreases expression and delays response to the growth factor stimuli (12, 38). Our findings suggest that chronic hyperglycemia adversely affects the epidermal barrier in children with diabetes. Long-term disturbances in glycemic control appear to compromise skin barrier integrity by impairing insulin and IGF-1 signaling in keratinocytes. To our knowledge, there are no previous studies evaluating TEWL in children with diabetes, which underscores the originality and significance of our research.

In our cohort, no associations were detected between TEWL or epidermal hydration and microvascular complications. However, given the small number of patients with complications and the relatively short disease duration in our study, these results should be interpreted with caution. Studies in adults with long-standing diabetes have reported associations between TEWL and corneometry values and diabetic complications, underscoring the need for long-term follow-up to better evaluate this relationship. Significant associations were observed between both TEWL and epidermal hydration and the presence of coexisting bronchial asthma. In children with asthma, TEWL values (p = 0.050; **Supplementary Table S3**) and epidermal hydration measurements (p = 0.001; **Figure 6**) were significantly lower than in children without asthma, suggesting a notable influence of atopy-related factors on these parameters, even after excluding patients with active atopic dermatitis. It should be noted that, in our study group, no correlation was observed between poorer metabolic control or the frequency of hyperglycemia and the presence of dry skin (data not presented; results on skin lesions in our cohort are currently being prepared for publication). This observation, along with the other findings reported, underscores the high sensitivity of biophysical measurements of the epidermal barrier and highlights their clinical utility. Dry skin in children with diabetes is often overlooked, yet these assessments are more sensitive, objective, and accurate, potentially enabling the identification of individuals at risk for neuropathy or the development of diabetic foot. In particular, regular skin care and the management of excessive body weight may improve epidermal barrier function and concurrently reduce the risk of skin infections in patients with diabetes (39, 40). Epidermal barrier dysfunction is associated with skin dysbiosis, which, together with an increased risk of skin breaches, elevates the susceptibility to skin infections and potentially contributes to the development of diabetic foot ulcers.

A key strength of the study protocol is the inclusion of healthy siblings of children with diabetes, allowing for partial control of environmental and genetic factors that may influence the observed outcomes. However, this approach contributed to the relatively small size of the control group. A notable limitation of the study was the reliance on existing medical records to obtain data on metabolic control, including HbA1c values, comorbidities, microvascular complications, and other risk factors. Future studies would benefit from incorporating up-to-date laboratory analyses and additional assessments in the pediatric population under investigation. This study did not include additional diagnostic procedures due to participants’ reluctance to undergo more medical interventions. During recruitment, the author frequently encountered objections from patients and their parents to invasive procedures, including blood withdrawals for laboratory analyses. Participation was contingent on the non-invasive nature of the assessments and minimizing the burden on both children with diabetes and their healthy siblings. Although parameters such as current glycemia during examination or cholesterol levels in the control group would have enhanced the study’s rigor, these measurements could not be performed. Nevertheless, the clinical data obtained are highly complete, reflecting comprehensive routine care and regular evaluation as part of standard diabetes management. Furthermore, the use of medical records allowed access not only to current HbA1c values but also to measurements obtained over the previous year, providing valuable insight into chronic metabolic control, which is particularly relevant for assessing the risk of complications (41–43). Conversely, using siblings of patients as the control group may be considered a limitation, as it resulted in a reduced control group size.

Clinicians should routinely monitor children with diabetes for dry skin, and non-invasive assessments, such as TEWL measurement and corneometry, may help identify individuals at risk for neuropathy or the development of diabetic foot complications. Early detection using these methods could facilitate targeted interventions, including enhancing patient motivation for optimal diabetes management, providing education on regular skin care, implementing hydration strategies, and promoting weight management, all of which may improve epidermal barrier function and reduce the risk of complications.

Further studies are needed to evaluate the utility of these techniques in greater detail. This project addresses an important and underexplored topic, and may be considered a pilot study that emphasizes the need for larger-scale research, particularly in light of the increasing use of continuous glucose monitoring systems. Our findings may improve the design of future studies and contribute to advancing understanding in this field. Longitudinal studies to monitor TEWL and corneometry in children with type 1 diabetes would help determine whether early abnormalities predict the development of complications such as neuropathy or diabetic foot. Additionally, future research could include larger and more diverse control groups, site-specific skin assessments, and integration with continuous glucose monitoring data. These steps will help validate the clinical utility of TEWL and corneometry as routine, non-invasive tools for early intervention and personalized diabetes care.

## 5 Conclusions

In our study, poor metabolic control in type 1 diabetes, reflected by higher HbA1c levels and more frequent hyperglycemia, was associated with alterations in TEWL and epidermal hydration. Additionally, body fat percentage (BF%) showed a positive correlation with TEWL. Incorporating supplementary assessments, such as TEWL measurement and corneometry, into routine examinations of children and adolescents with type 1 diabetes is recommended. Clinicians should remain vigilant for dry skin in this population, as noninvasive evaluations may help identify individuals at increased risk of neuropathy or diabetic foot development. Further studies are warranted to comprehensively assess the clinical utility of TEWL and corneometry in this context.

## Data Availability

The raw data supporting the conclusions of this article will be made available by the authors, without undue reservation.
