# Supplementary material for: Selected parameters of epidermal barrier in juveniles with type 1 diabetes correspond with the severity of diabetes – an observational study
Source: Front Endocrinol (Lausanne). 2025 Nov 25;16:1709604. doi: 10.3389/fendo.2025.1709604 (PMC12685653; doi:10.3389/fendo.2025.1709604)
Supplement: Supplementary file 1 [file Table1.docx]

# SUPPLEMENT MATERIAL

Table S1. Correlations between the TEWL value and basic clinical parameters in Study Group A

|  |  | **Age**  **(years)** | **Diabetes duration (years)** | **BMI**  **SDS** | **BF(%)** | **Skinfold arm**  **(mm)** | **Skinfold abdomen**  **(mm)** | **Skinfold subscapular**  **(mm)** |
| --- | --- | --- | --- | --- | --- | --- | --- | --- |
| **TEWL (g/m2/h)** | R_S_  *p* | 0,042  0,654 | 0,059  0,524 | 0,072  0,439 | 0,214  0,025 | 0,137  0,157 | 0,151  0,117 | 0,164  0,089 |

R_S_ – Spearman’s correlation coefficient, TEWL – transepidermal water loss, BF(%) – body fat mass

Among the features examined in this section of the results, one statistically significant difference was found - the BF level correlated positively with TEWL. There was no correlation between clinical parameters like age, disease duration, BMI-SDS or skinfolds thicknesses and TEWL.

Table S2. Correlations between the TEWL value and clinical parameters concerning metabolic control in study group A

|  |  | **HbA_1c_ (%) last** | **HbA_1c_ (%) mean** | **Hyperglycemia frequency** | **Hypoglycemia frequency** | **DID U/kg** |
| --- | --- | --- | --- | --- | --- | --- |
| **TEWL (g/m2/h)** | R_S_  *p* | 0,183  0,052 | 0,195  0,043 | 0,077  0,414 | 0,011  0,907 | 0,005  0,960 |

R_S_ – Spearman’s correlation coefficient, TEWL – transepidermal water loss, HbA_1c_ (%) last – glycated hemoglobin – mean from results taken in three months, HbA_1c_ (%) mean – glycated hemoglobin, mean from results taken in last year, DID – daily insulin dose in units per kilograms of body weight

Among the features examined in this section of the results, one statistically significant difference was found – mean HbA1C (%) correlated positively with TEWL, which means that patients with higher HbA1C had higher transepidermal water loss. There was no correlation between clinical parameters concerning insulin treatment or glycemic levels and TEWL.

Table S3. Analysis of the relationship between the TEWL value and the presence of comorbidities

|  |  | **TEWL (g/m2/h)**  **Mean ± SD** |  | **TEWL (g/m2/h)**  **Mean ± SD** | ***p*** |
| --- | --- | --- | --- | --- | --- |
| **Autoimmune thyroidis** | **yes** | 0,18 ± 0,03 | **no** | 11,41 ± 3,20 | 0,092 |
| **Celiac disease** | **yes** | 11,05 ± 5,11 | **no** | 11,00 ± 3,50 | 0,844 |
| **Asthma** | **yes** | 11,26 ± 5,06 | **no** | 8,73 ± 2,42 | 0,050 |
| **Vitiligo** | **yes** | 11,03 ± 4,98 | **no** | 11,44 ± 4,38 | 0,741 |

TEWL – transepidermal water loss, SD – standard deviation, p-level of significane < 0,05; U Mann-Whitney test

Among comorbidities, one statistically significant difference was found, patients with asthma had lower TEWL, although the significance of the result was low.

Table S4. Correlations between corneometry results and clinical parameters regarding metabolic control in Study Group A

|  |  | **HbA_1c_ (%) last** | **HbA_1c_ (%) mean** | **Hyperglycemia frequency** | **Hypoglycemia frequency** | **DID U/kg** |
| --- | --- | --- | --- | --- | --- | --- |
| Corneometry (U) | R_P_  *p* | -0,023  0,808 | 0,018  0,850 | -0,212  0,022 | 0,041  0,659 | -0,005  0,961 |

R_P_ – Pearsons coefficient, HbA_1c_ (%) last – glycated hemoglobin – mean from results taken in three months, HbA_1c_ (%) mean – glycated hemoglobin, mean from results taken in last year, DID – daily insulin dose in units per kilograms of body weight

Among the features examined in this section of the results, one statistically significant difference was found – hyperglycemia frequency correlated negatively with corneometry, which means that patients with higher frequency of hyperglycemia had lower epidermis hydration, which is presented on the Figure 3 in the main text. There was no correlation between clinical parameters concerning HbA1c, insulin treatment or glycemic levels and corneometry .

Table S5. Analysis of the relationship between the corneometry results and the presence of comorbidities

|  |  | **Corneometry (U)** Mean ± SD |  | **Corneometry (U)**  Mean ± SD | ***p*** |
| --- | --- | --- | --- | --- | --- |
| **Autoimmune thyroidis** | **yes** | 36,58 ± 9,75 | **no** | 34,06 ± 10,36 | 0,237 |
| **Celiac disease** | **yes** | 36,41 ± 10,11 | **no** | 34,52 ± 10,26 | 0,519 |
| **Asthma** | **yes** | 24,84 ± 9,47 | **no** | 35,67 ± 9,82 | 0,001 |
| **Vitiligo** | **yes** | 37,53 ± 6,17 | **no** | 34,62 ± 10,36 | 0,660 |

SD – standard deviation

Among comorbidities, one statistically significant difference was found, patients with asthma had lower corneometry results, which means that patients with asthma had lower epidermal hydration.
